# Superior Proton Exchange Membrane Fuel Cell (PEMFC) Performance Using Short-Side-Chain Perfluorosulfonic Acid (PFSA) Membrane and Ionomer

**DOI:** 10.3390/ma15010078

**Published:** 2021-12-23

**Authors:** Nana Zhao, Zhiqing Shi, Francois Girard

**Affiliations:** Energy, Mining & Environment Research Centre, National Research Council Canada, 4250 Wesbrook Mall, Vancouver, BC V6T 1W5, Canada; Nana.Zhao@nrc-cnrc.gc.ca (N.Z.); Francois.Girard@nrc-cnrc.gc.ca (F.G.)

**Keywords:** ionomer, long side chain, membrane, perfluorosulfonic acid, PEM fuel cell, short side chain

## Abstract

Optimization of the ionomer materials in catalyst layers (CLs) which sometimes is overlooked has been equally crucial as selection of the membranes in membrane electrode assembly (MEA) for achieving a superior performance in proton exchange membrane fuel cells (PEMFCs). Four combinations of the MEAs composed of short-side-chain (SSC) and long-side-chain (LSC) perfluorosulfonic acid (PFSA) polymers as membrane and ionomer materials have been prepared and tested under various temperatures and humidity conditions, aiming to investigate the effects of different side chain polymer in membranes and CLs on fuel cell performance. It is discovered that SSC PFSA polymer used as membrane and ionomer in CL yields better fuel cell performance than LSC PFSA polymer, especially at high temperature and low RH conditions. The MEA with the SSC PFSA employed both as a membrane and as an ionomer in cathode CL demonstrates the best cell performance amongst the investigated MEAs. Furthermore, various electrochemical diagnoses have been applied to fundamentally understand the contributions of the different resistances to the overall cell performance. It is illustrated that the charge transfer resistance (R_ct_) made the greatest contribution to the overall cell resistance and then membrane resistance (R_m_), implying that the use of the advanced ionomer in CL could lead to more noticeable improvement in cell performance than only the substitution as the membrane.

## 1. Introduction

Proton exchange membrane fuel cells (PEMFCs) have been widely considered to be a critical conversion technology in a hydrogen-based energy infrastructure due to their high theoretical energy efficiency and zero-emission [1,2]. The proton exchange membrane (PEM) functioning as a proton conductor as well as a separator for electrodes and reactant gas was recognized as one of the most expensive stack component and a key component to determine the cell performance [3]. On the other hand, the microstructure of electrode has a profound effect on the utilization of platinum and its durability [4]. Particularly, the ionomer in the catalyst layer can affect the degree of ionic contact, the connectivity of the ionic conduction path, the proton conductivity, the gas diffusivity, etc. Therefore, the proton exchange polymers employed in PEMFC as membrane and as binder/proton conductor in the catalyst layer (CLs) are equally crucial to the entire cost and performance of a PEMFC.

Perfluorosulfonic acid (PFSA) polymers have been widely used as PEMs and as ionomers in CLs. Currently, the premiere commercial PFSA used in PEMFCs is Nafion^®^, a long-side-chain (LSC) ionomer, produced previously by DuPont and now Chemours, which is a brand name for a sulfonated tetrafluoroethylene based fluoropolymer discovered in the late 1960s by Walther Grot of DuPont [5]. The Nafion polymer structure consists of a hydrophobic polytetrafluoroethylene backbone and perfluorovinyl ether side chains terminated by a triflic acid group (-CF_2_SO_3_H) [6]. Although Nafion™ possesses many desirable attributes, it has the recognized limitation of insufficient proton conductivity under low humidity and a limited range of operating temperature (<90 °C). Furthermore, the proton conductivity depends on the ratio and phase separation of hydrophobic backbone and hydrophilic side chains [7]. The short-side-chain (SSC) PFSA polymer with a similar structure as Nafion, but bearing a shorter -OCF_2_CF_2_SO_3_H pendant chain, has been considered as a promising candidate for PEMFC applications due to the higher crystallinity, the higher thermal transition temperature, and the higher ion exchange capacity (IEC) compared to LSC ionomer [8]. SSC polymer was originally synthesized by Dow Chemical [9] and its superior fuel cell performance was firstly demonstrated by Ballard Power Systems in the mid-1980s [10]. In 2010, Solvay-Solexis developed a simpler approach to synthesize SSC PFSA polymers under the trademark “Aquivion^®^”, reporting higher IEC value, higher water uptake, similar mechanical properties, higher glass transition temperature, and comparable price with respect to Nafion [11].

A number of research reports have demonstrated the distinct advantage of SSC PFSA ionomers in fuel cells [12,13,14,15,16,17,18,19,20,21,22]. It was reported that the SSC PFSA membranes exhibited higher fuel cell performance than Nafion under low humidity as well as higher power density, better fuel cell reliability, and settling time upon start-up in self-humidifying PEMFCs under the optimal operating conditions [14,18]. With the incorporation of the SSC PFSA ionomer only in CLs, an increase in cell voltage was observed compared to the cell with Nafion as ionomer [12,13,19,20,21]. Arico’s group [15] compared the fuel cell performance between Aquivion^®^ E79-03S SSC (130 °C) and LSC Nafion^®^ PFSA membranes as baselines at a high temperature and found the Aquivion-based membrane electrode assembly (MEA) showed better performance due to their intrinsic properties of SSC such as the larger crystallinity, higher glass transition temperature, and higher IEC. Shahgaldi et al. [7] experimentally investigated the impact of different catalyst-SSC ionomer ratios and catalyst loadings on PEMFC performance and durability with an accelerated stress test. Ren et al. [21] reported that the electrode composed of SSC ionomer has better proton conductivity but higher mass transport resistance than Nafion, which is still superior in the ultimate performance. Although considerable efforts have been devoted to investigating SSC PFSA polymer either as a membrane or as a binder in CLs, reporting the comparative studies on the combination of SSC and LSC PFSA as a membrane and as an ionomer in CLs to achieve the best PEMFC performance are not-so-well documented. More recently, Talukdar’s group [17] compared performance and durability between LSC and SSC PFSA polymers as a membrane and as an ionomer-additive in the electrodes, whereas they were focused on the short-term and long-term durability test. In their short-term test, they found SSC has better performance but higher degradation rate than LSC. In the long-term test, the cell durability was improved by increasing the membrane thickness with double SSC membranes. Furthermore, it is commonly agreed that the SSC polymer as either a membrane or ionomer in CL can usually deliver higher performance than the LSC polymer does. The question of whether the SSC membrane or the SSC ionomer impacts more on fuel cell performance than the LSC polymer has not been addressed.

In our present work, a comprehensive study is carried out to compare the fuel cell performance between the MEAs with a combination of both SSC and LSC polymers in the membranes as well as the ionomers in CL under various operation conditions in order to understand the correlations between the MEA composition and fuel cell performance. The complicated influence of polymers with different side chain lengths on PEMFC performance was addressed by means of in-situ EIS and the analysis of the relative contribution of individual resistance, including membrane resistance, charge transfer resistance and mass transfer resistance, to the overall resistance generated during fuel cell testing. The understanding of the role of each component in MEAs from this work is essential to optimize and improve fuel cell performance and could provide deep insight regarding polymer material selection and MEA design for PEMFC applications.

## 2. Materials and Methods

### 2.1. Materials

Nafion^®^ NR211 membrane (NRE211, 25 µm LSC PFSA, DuPont, DE, USA) and Aquivion^®^ E79-03S membrane (AQ(E79), 30 μm, SSC PFSA, Solvay-Solexis) were used as-received. The Nafion^®^ LSC ionomer dispersion (D-520, 5 wt% solution, EW1100, DuPont) and the Aquivion^®^ SSC ionomer dispersion (D83-15C, 15 wt% solution, EW 830, Solvay Solexis) were used as the ionomers in CLs.

### 2.2. Fabrication of Membrane-Electrode-Assemblies (MEAs)

Catalyst inks were prepared by dispersing 46 wt% Pt/C (Tanaka Kikinzoku Kogyo, Tokyo, Japan) with ionomer into the mix solvent of 1:1 methanol/water. The solid content of the inks was ~1 wt%. The Nafion^®^ D-520 ionomer content in the cathode CL was 30 wt% while the Aquivion^®^ D83-15C ionomer content in the CL was 20 wt%. The anode CL for all MEAs contained 30 wt% Nafion^®^ D-520 ionomer. An automated spray coater (EFD Ultra TT series) was employed to fabricate the anode CL (0.2 mg Pt cm^−2^) and the cathode CL (0.4 mg Pt cm^−2^). The active area of CCMs is 25 cm^2^. Detailed fabrication procedures have been reported [14,23]. The investigated MEAs are listed in Table 1.

### 2.3. MEAs and Single Cells

CCMs were inserted between two 24BC-type gas diffusion layers (SGL Group) to fabricate MEAs (see Figure S1) and assembled into single cells (Scribner Associates Inc. Southern Pines, NC, US). The uniformity of the cell compression was validated by the pressure-sensitive films (Pressurex-Super Low, Sensor Products Inc. Madison, WI, USA). Single cells were evaluated in a fuel cell test station (100 W, Scribner 850C, Scribner Associates Inc. Southern Pines, NC, USA).

### 2.4. Fuel Cell Testing Protocol

The MEAs were conditioned at 1.0 A cm^−2^, 100% relative humidity (RH), 80 °C for at least 10 h. Air and H_2_ (purity 99.999%) were used as the cathode and anode reactants with 5 standard liter per minute (SLPM) and 2 SLPM of flow rate, respectively. The humidifiers are dual sparger-type with 360 W heaters per bottle. The membrane resistance (R_Membr_.) was measured by current interrupt method.

Electrochemical surface area (ECSA) was measured by cyclic voltammetry (CV) using a potentiostat (1287A, Solartron Analytical). The cathode were purged with humidified N_2_ (0.5 SLPM) for 30 min. The N_2_ flow was then set to zero and H_2_ flow rate keeps 0.5 SLPM, and voltammograms were then recorded with a scan rate of 50 mV·s^−1^ between 0.04 V and 0.90 V versus the anode. The final cycle of a set of 10 cycles was used for data analysis. The ECSAs of the MEAs were calculated from the integrated charge corresponding to the Pt-H desorption and adsorption peaks. The double-layer capacitances of the CLs were also obtained from CV.

The H_2_ cross-over current density was measured by linear sweep voltammetry (LSV) with a scan rate of 2 mV s^−1^ between 0.1 V and 0.6 V versus the anode under 0.5/0.5 SLPM H_2_/N_2_ (anode/cathode) gas flow rates.

The ionic resistance in the CLs was evaluated by ex-situ EIS using a Solartron 1287A potentiostat and a 1260 frequency response analyzer. The testing was conducted with the flow rates of 0.5 SLPM/0.5 SLPM for H_2_ and N_2_, respectively, at 100% RH, and 80 °C. The amplitude of the sinusoidal current signal for the AC impedance was set at 10 mV over a frequency range of 20 kHz to 0.1 Hz.

The ORR kinetics of these CLs were determined by acquiring H_2_/O_2_ polarization curves at gas flow rates of 2 SLPM H_2_ and 5 SLPM O_2_ without back pressure. The current load was gradually decreased in controlled steps from 1 A cm^−2^ to 0.008 A cm^−2^. Each point was held for 10 min. The cell voltage at each current was obtained by averaging the data recorded in the last 2 min. The *iR*-corrected cell potentials were plotted against the log of compensated current density, and the Tafel slope was extracted.

The cell performances of these MEAs were evaluated at 80 °C and 95 °C under different RHs (100%, 70%, 50%, and 30%) for both anode and cathode without back pressure. The H_2_/air polarization curves were obtained galvanostatically. Each point was held for 10 min. The cell potential was obtained by averaging the data from the last 2 min.

The *iR* correction to voltages were calculated by the Equation (1):

(1) $$E_{iR\;correction}=E_{Measured}+iR_{Membr.}$$

where $E_{iR\;correction}$ and $E_{Measured}$ represent *iR* correction cell voltage and the cell voltage measured in H_2_/air polarization curve, respectively. i is the current and $R_{Membr.}$ is the membrane resistance collected by the current interrupt method.

In-situ EIS was conducted during polarization curve collections (under constant direct current (DC)) by imposing an amplitude alternating current (AC) signal to the fuel cell via a load bank. The perturbation amplitude for the AC impedance was 5% of the direct current over a frequency range of 10 kHz to 0.1 Hz. The voltage responses were recorded and decoupled by a built-in frequency response analyzer (FRA, Scribner 880).

## 3. Results

### 3.1. Electrochemical Surface Area (ECSA)

**Figure 1:**
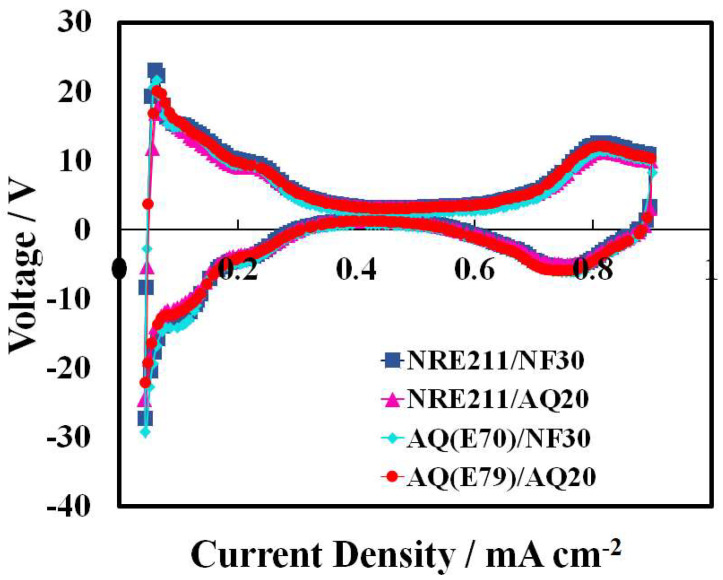
Typical cyclic voltammograms of cathode CLs in MEAs at 80 °C and 100% RH.

The ECSA of Pt catalyst in the cathode was evaluated by cyclic voltammetry (CV). Figure 1 shows the CVs of different CLs in MEAs at 80 °C and 100% RH. The Nafion^®^-based CLs (NRE211/NF30 and AQ(E79)/NF30) exhibit characteristic features of hydrogen adsorption/desorption and oxide formation/reduction that are similar to the Aquivion^®^-based CLs (NRE211/AQ20 and AQ(E79)/AQ20). ECSA data and double layer capacitances (C_dl_) for the selected four MEAs are also presented in Table 2. The ECSA from desorption peaks (voltage range from 0.11 V to 0.40 V) and C_dl_ values were close for all the samples, between 37.5 and 39.6 m^2^ g^−1^ and 19.0 and 20.3 mF cm^−2^, respectively, indicating that the availabilities and coverage of Pt nanoparticles by the LSC ionomer and SSC ionomer are similar.

### 3.2. Hydrogen Crossover Current

Hydrogen crossover currents (iH_X_) through the MEAs were detected by LSV. The oxidation current densities at 80 °C and 100% RH were found to be less than 1.6 mA cm^−2^ for all the MEAs (see Table 2). These low crossover currents attributed to less than 2% of the current density of a fuel cell operating, e.g., 0.8–0.3 V, 0.1–1.8 A cm^−2^, (see polarization curves below), suggesting that the efficiency loss is negligible due to the H_2_ crossover from anode to cathode in these MEAs. In addition, the crossover currents are nearly the same for the two SSC membrane-based MEAs (~1.2 mA cm^−2^), while they are slightly lower than that of LSC Nafion based MEAs (~1.6 mA cm^−2^), indicating that the differences between these MEAs in H_2_ crossover are dictated only by the membrane, and thus SSC AQ(E79) has a lower hydrogen crossover than LSC NRE-211. The lower H_2_ crossover for SSC AQ(E79) could possibly be elucidated by the free volume theory from Sodaye and Mohamed et al. [24,25]. SSC AQ(E79) has higher crystallinity, greater IEC (1.26 vs. 0.9 meq kg^−1^) and better water uptake than Nafion [11,26], leading to the greater volume of the hydrophilic cluster in the hydrated AQ(E79) than that in NRE211, as a consequence, the less free volume for the hydrogen crossover [27].

### 3.3. Protonic Resistance in the CL

**Figure 2:**
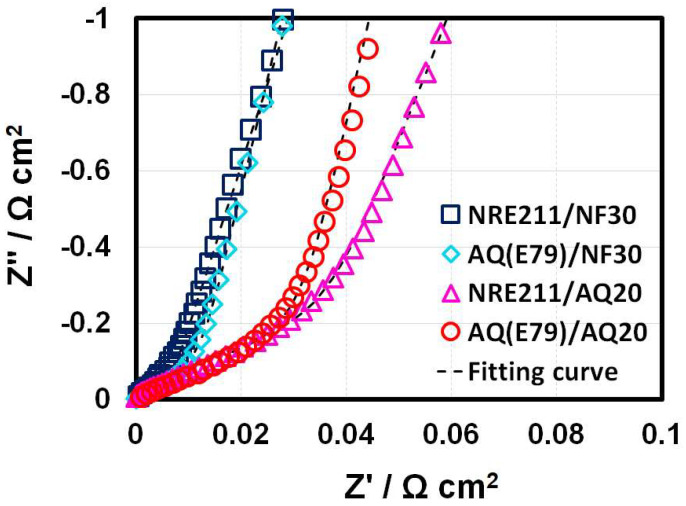
Nyquist plots of cathode CLs in the four MEAs under 80 °C and 100% RH.

The proton conductivities of the CLs were measured by EIS at 80 °C and 100% RH. Impedance spectra of four MEA samples under this test condition are presented as Nyquist plots in Figure 2, respectively. In order to make clear, the spectra are shifted to the origin by removing the high-frequency resistance from the real axis. The protonic and electronic resistances inside the CLs were calculated by fitting the experimental data by a transmission line equivalent circuit [23]. The proton resistance (R_p_) values are also summarized in Table 2.

As a result, there are no obvious differences in protonic resistance (or conductivity) between either Nafion^®^-electrodes (NF30) or Aquivion^®^-electrodes (AQ20) due to the similar coverage of carbon surface with fully hydrated ionomers at 100% RH. However, in comparison between NF30 based MEAs and AQ20 based MEAs, AQ20 exhibited a greater proton transport resistance (lower proton conductivity) of the CLs due to their relatively lower ionomer content, while at the same ionomer loading (30%), AQ30 was reported to have a lower proton resistance (higher proton conductivity) of the CL in a previous report [12]. Generally, the ionomer loading in the CLs has a conflict between the proton conductivity and the porosity for reactant transport (e.g., gas, water) since overloaded ionomer in the CLs reduces the porosity while inadequate ionomer lowers the proton conductivity, respectively [7]. Shahgaldi et al. reported that the SSC ionomer is less sensitive to its loading with the ionomer ratios in the range between 17% and 30% due to the high uniformity coverage of Pt nanoparticles [28]. It was reported that 20 wt% of SSC Aquivion^®^ D83-15C ionomer content in CLs exhibited the best performance under operating conditions at 95 °C and RH values of 30%, 50%, and 70% [12]. Therefore, 20% of the SSC ionomer loading was selected in this work. In addition, the studies revealed that the optimal Nafion^®^ loading in the CL was ∼30 wt% [29,30].

### 3.4. ORR Kinetics

**Figure 3:**
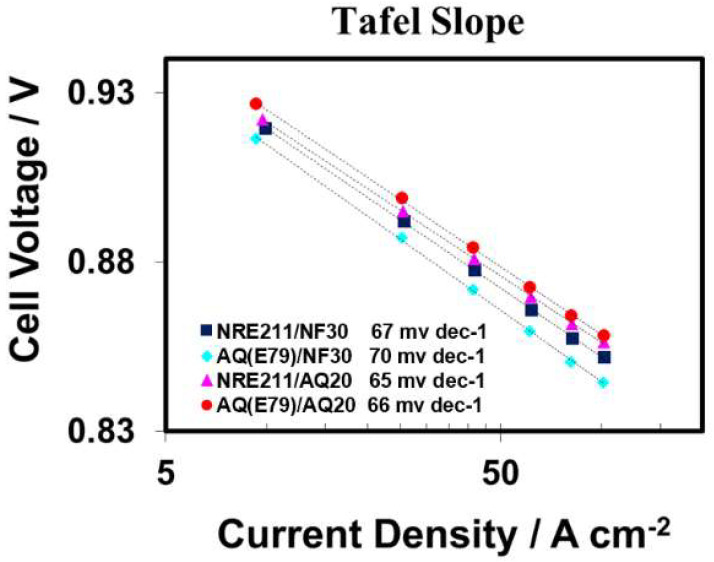
Tafel plots of H_2_/O_2_ polarization at 80 °C and 100% RH for all investigated MEAs.

H_2_/O_2_ polarization curves (PLs) of the four MEAs were recorded at 80 °C and 100% RH with excessive gas flow rates to elusive the losses associated with mass transport in the ORR kinetic range. Figure 3 shows the Tafel plots extracted from PLs using the *iR*-corrected electrode potentials (defined as the measured potential plus the *iR*) against the logarithm of the H_2_-crossover compensated current density (defined as the measured current density plus the iH_X_). In the kinetic region (E > 0.80 V), the average Tafel slope for the MEAs with 30 wt% of Nafion^®^ (NF30) (~68.5 mv dec^−1^) were almost the same as the average Tafel slope of the MEAs with 20 wt% Aquivion^®^ D83-15C (AQ20) (65.5 mv dec^−1^). This similarity implied that these four different MEAs demonstrated the similar inherent Pt/C electrode kinetics towards ORR because the Pt catalyst coverage is uniform with sufficiently hydrated ionomer at 100% RH [31]. The slightly higher Tafel slope of AQ(E79)/NF30 (70 mVdec^−1^) probably can be attributed to the incompatibility of Nafion^®^ ionomer with Aquivion^®^ membrane [16].

**Figure 4:**
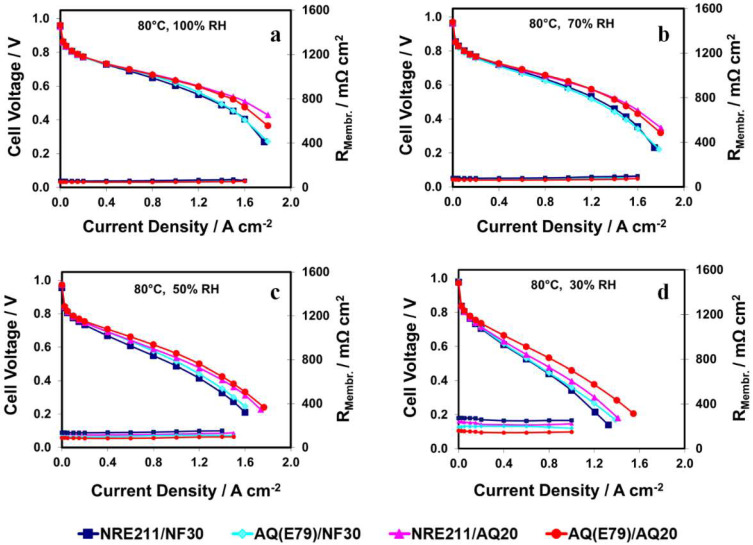
H_2_/air polarization curves and membrane resistance (R_Membr._) at 80 °C and RH values of (**a**) 100%; (**b**) 70%; (**c**) 50%; and (**d**) 30%.

The H_2_/air polarization curves (PLs) collected at 80 °C and various RHs, as well as the membrane’s ionic resistance (R_Membr_., predominate contribution to the cell ohmic resistance), are plotted in Figure 4. Three pieces of NRE211/NF30 were tested and used to establish the reproducibility of the MEA fabrication and testing procedures and their average PLs are shown in Figure 4. In the kinetic region (current densities < 0.1 A cm^−2^), the cell performances for all MEAs under various operation conditions were close (a difference in potential of <10 mV at 0.1 A cm^−2^), which is in agreement with the Tafel slope analysis shown in Figure 3.

**Figure 5:**
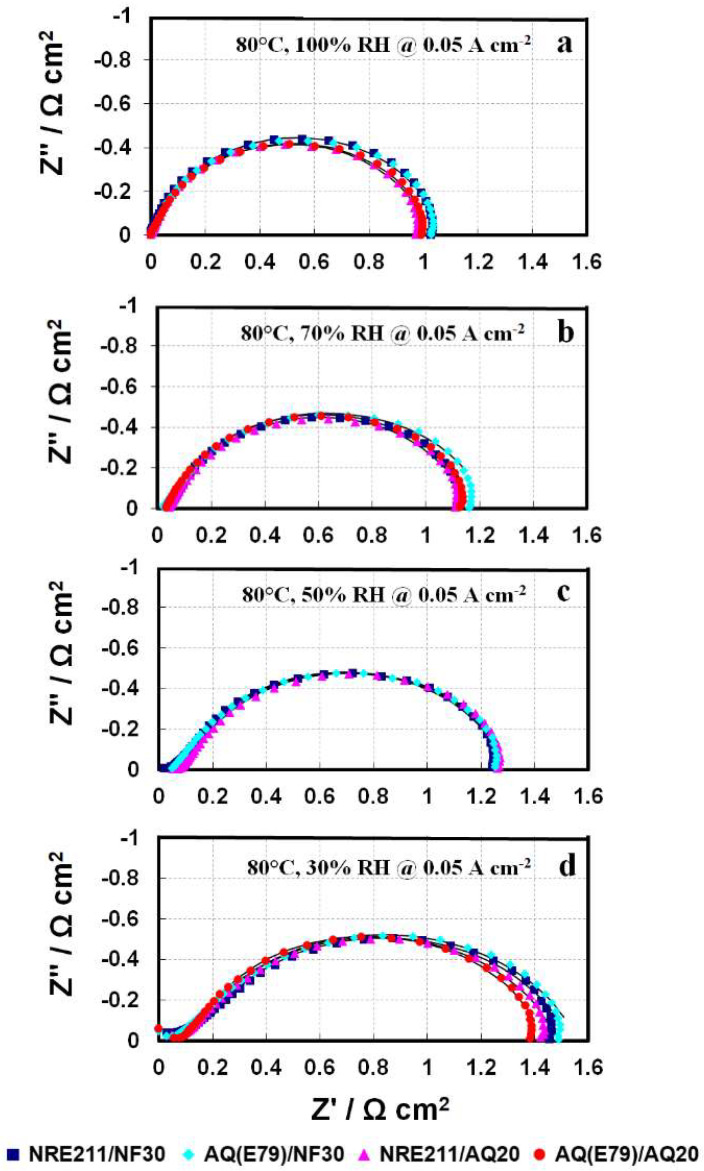
In-situ electrochemical impedance spectra obtained at 80 °C, 0.05 A cm^−2^, and (**a**) 100%; RH (**b**) 70% RH; (**c**) 50% RH; and (**d**) 30% RH.

In-situ EIS could provide some valuable insights into transport processes occurring in the MEAs. Figure 5 shows the in-situ EIS at 0.05 A cm^−2^ for the selected MEAs under various RH. At low current densities (e.g., 0.05 A cm^−2^), it is assumed that there are negligible losses associated with the transport of reactant gases to the electrode reactive sites since the oxygen consumption rate is small. For better comparison, the high-frequency intercepts are offset to zero. In Figure 5, each spectrum contains one small high-frequency (HF) capacitive arc and one big capacitive arc (1000–1 Hz). The small HF loop is almost negligible with respect to the big single semicircle (kinetic loop) [32]. Thus, only the kinetic loop was analyzed and fitted by a charge transfer resistance (R_ct_) in parallel with a constant phase element (CPE). The fitting curves (black solid lines) are shown in Figure 5.

The difference in R_ct_ for these four MEAs at each operation condition is slight, matching with the trend of PL curves in Figure 4. It implies that the similar ORR kinetics in these four samples can be associated with a comparable resistance, R_ct_. Moreover, as the RH changes from 100% to 30%, the R_ct_ in Figure 5 for all the samples gradually increased from 1.04 ± 0.02 Ω cm^2^ to 1.37 ± 0.06 Ω cm^2^, along with the cell voltage drop from 806 ± 3 mV to 770 ± 6 mV in Figure 4, indicating that the reduced ORR kinetics (cell performance) at “dry” condition (RH 30%) is associated to the larger resistance, R_ct_. With decreasing RH, the R_ct_ increases due to the dehydration of ionomer in both the membrane and catalyst layers, leading to proton conductivity losses and inferior catalyst-ionomer reactive interphase [16].

### 3.5. Mass Transfer Losses

By contrast, in the mass transport region (current densities ≥1.0 A cm^−2^) of the PL curves shown in Figure 4, the cell performances for the MEAs containing Aquivion^®^ ionomer (AQ20) were consistently greater than that of MEAs containing Nafion^®^ ionomer (NF30) and the voltage gap between them becomes even broader when either the current density increases from 1.0 A/cm^2^ to 1.8 A/cm^2^ or RH decreases from 100% to 30%. For example, comparing between NRE211/NF30 and NRE211/AQ20 at 80 °C and 70% RH, the difference in cell voltage increased from ~30 mV at 1.0 A cm^−2^ to ~100 mV 1.6 A cm^−2^. Furthermore, in comparison to NF30 based MEAs, the performance improvement of AQ20 based MEAs, was more pronounced at low RH.

**Figure 6:**
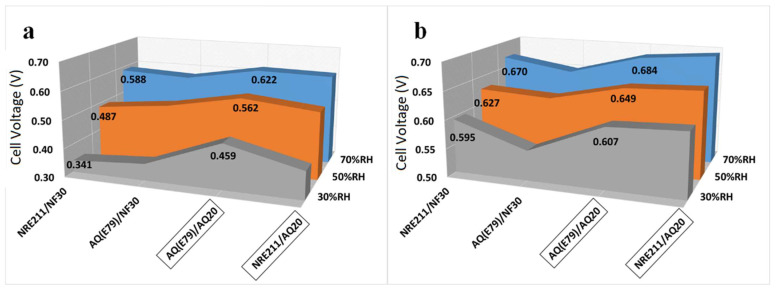
(**a**) Cell voltage without *iR* correction; (**b**) cell voltage with *iR* correction at a current density of 1.0 A cm^−2^ at 80 °C and various RH.

Figure 6a shows the cell voltage at a current density of 1.0 A cm^−2^ at 80 °C and various RH. The maximum cell voltage differences between these four samples increased from 46 mV to 118 mV after RH was reduced from 70% to 30%, indicating the advantage of SSC membrane/ionomer was more prominent under low RH.

In order to count out the effect of the membrane resistance (R_Membr_.) on the PLs, *iR*-compensated H_2_/air polarization curves were calculated using R_Membr_., as shown in Figure S2. The *iR*-compensated cell voltages at a current density of 1.0 A cm^−2^ are displayed in Figure 6b. After subtracting R_Membr_., the MEAs containing 20% of SSC AQ ionomer (AQ20) exhibited higher cell performance than the MEAs containing 30% of LSC Nafion ionomer (NF30), which is more pronounced under low RH (30%) (Figure 6b). For example, the difference in cell voltage at 1.0 A cm^−2^ between AQ (E79)/NF30 and AQ (E79)/AQ20 increased from 35 mV to 55 mV when RH decreased from 70% to 30%, revealing that the higher performance of AQ20 ionomer based MEAs over NF30 ionomer based MEAs can partially be attributed to the advantage of SSC ionomer in the catalyst layer.

Moreover, when comparing the cell voltage with and without *iR* correction at 1.0 A cm^−2^ shown in Figure 6, the contribution of SSC membrane to the superior cell performance is distinguishable, especially at low RH. For example, under conditions of 30% RH, the cell voltages with *iR* correction for AQ(E79)/AQ20 and NRE211/AQ20 are almost identical while the cell voltage without *iR* correction for AQ(E79)/AQ20 is over 60 mV higher than that for NRE211/AQ20.

**Figure 7:**
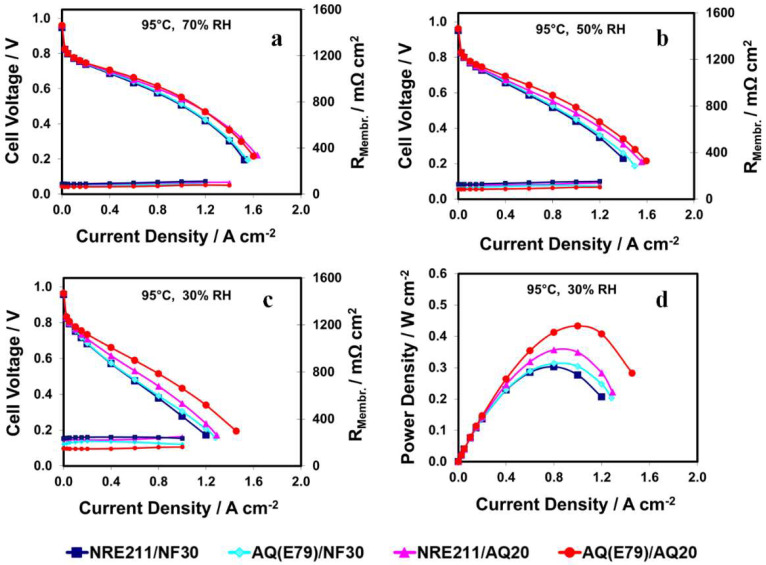
H_2_/air polarization curves and membrane resistance (R_Membr_.) at 95 °C and RH values of (**a**) 70%, (**b**) 50%, (**c**) 30%, as well as power densitites at RH of (**d**) 30%.

The same trend at high temperature (95 °C) was remarkably observed as above: increasingly higher relative performance for AQ20 based cells as RH was decreased (see Figure 7). This is more apparent when the RH was <50%. For example, under 30% RH a potential of 433 mV was generated for AQ(E79)/AQ20 at 1.0 A cm^−2^ in comparison to 277 mV for NRE211/NF30, representing a 56% increment in power output. Of the four investigated MEAs under 30% RH, AQ (E79)/AQ20 displayed the highest power density (0.43 W cm^−2^, see Figure 7d), followed by NRE211/AQ20 (0.36 W cm^−2^), and then AQ (E79)/NF30 (0.31 W cm^−2^) and NRE211/NF30 (0.30 W cm^−2^).

### 3.6. Cell Resistance

**Figure 8:**
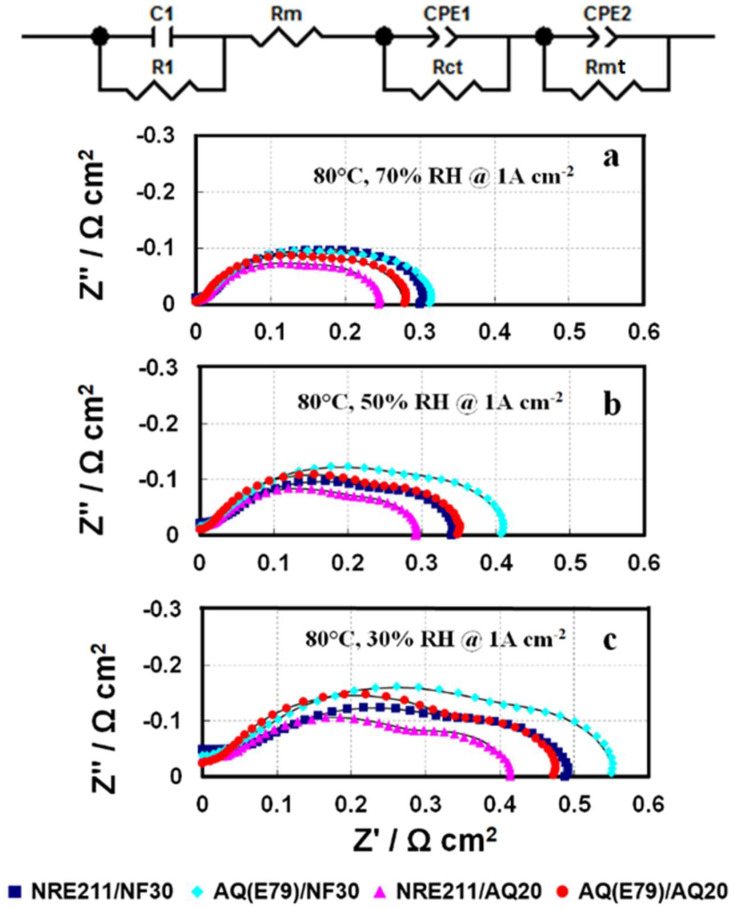
In-situ electrochemical impedance spectra of four investigated MEAs at a current density of 1.0 A cm^2^ at 80 °C under RH of (**a**) 70%; (**b**) 50%; and (**c**) 30%.

**Figure 9:**
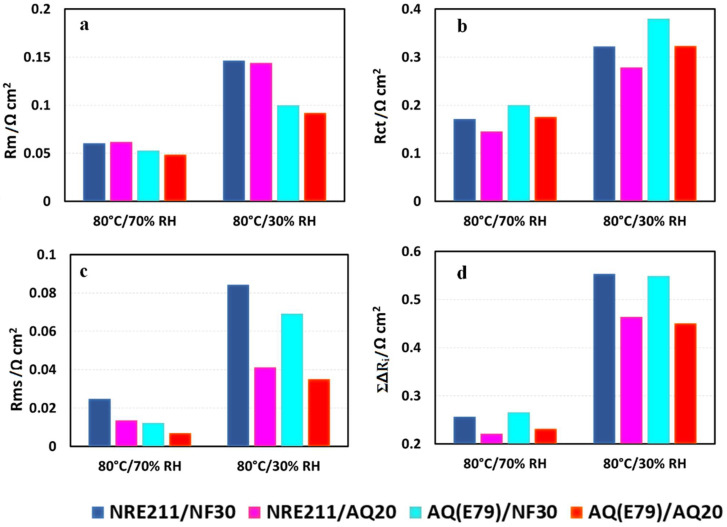
(**a**) Membrane resistance (R_m_); (**b**) charge transfer resistance (R_ct_); (**c**) mass transfer resistance (R_mt_); and (**d**) overall resistances (ΣΔR_i_) of each MEA calculated from EIS data at 80 °C under 70% and 30% of RH.

In order to further understand the observed behaviors of these four MEAs at various operating conditions, in-situ EIS were obtained for the MEAs at 1.0 A cm^−2^ shown in Figure 8. The Nyquist plots of the tested MEAs contains one high-frequency (HF) capacitive loop, one medium-frequency (MF) capacitive loop, and one low-frequency (LF) capacitive loop. For comparison, the high-frequency intercepts are offset to zero. The charge transfer resistance (R_ct_), membrane resistance (R_m_), and mass transfer resistance (R_mt_) were fitted using the equivalent circuit [33] shown in Figure 8, and the experimental section in supporting materials. The fitting curves (solid line) and fitting results are presented in Figure 8 and Figure 9, respectively.

#### 3.6.1. Membrane Resistance

Membrane resistance primarily contributes to the cell ohmic resistance. The trend for R_m_ from EIS fitting in Figure 9a is well-matched to the membrane resistance (R_membr_.) collected by the current interrupt technique in Figure 4: SSC AQ(E79) membrane-based cells yielded lower membrane resistances (R_membr_./R_m_) and higher conductivities compared to LSC NRE-211membrane-based cells under all fuel cell operating conditions. Among the four samples, AQ(E79)/AQ20 exhibits a minimum membrane resistance (R_membr_./R_m_) and a corresponding maximum conductivity (See Table 3). This trend is more significant when RH is reduced, which is linked to the inherent properties of the SSC polymer such as high IEC, high crystallinity and high water content.

#### 3.6.2. Charge Transfer Resistance

The effective charge transfer resistance (R_ct_) represents in the medium frequency domain in EIS associated with the ORR kinetics of the CL. AQ20 ionomer in CLs presents lower R_ct_ than NF30 ionomer under both 70% RH and 30% RH that led to the superior performance of the SSC AQ ionomer in CLs over that of the LSC Nafion ionomer. The improvement in the cell performance for the MEAs with SSC ionomer in CLs could be on account of better ionomer coverage in the CLs, superior proton conductivity, less equivalent weight, and greater porosity [7]. It is worth mentioning that AQ(E79)/AQ20 shows the best performance amongst the four samples, while the R_ct_ of this sample is still slightly higher than NRE211/AQ20, indicating that the outstanding performance of AQ(E79)/AQ20 benefits from the advantages of better compatibility of SSC membranes and SSC ionomer in CLs. We hypothesized that the marginally greater R_ct_ for AQ(E79)/AQ20 than NRE211/AQ20 (0.323 Ω cm^2^ vs. 0.279 Ω cm^2^ under 30% RH) is due to some possible deleterious effects from the combination of the high water content of the Aquivion^®^ membrane and ionomer: (i) dilution of the local proton concentration associated with swollen membrane and ionomer, (ii) decline of the oxygen solubility in the ionomer because of the high hydrophilicity of SSC ionomer, and (iii) relative high interfacial resistance between membrane and CL due to considerable SSC membrane dimensional change under fuel cell operation conditions.

#### 3.6.3. Mass Transfer Resistance

Mass transfer resistance (R_mt_) displays in the low frequency domain in EIS that mainly responses for the mass transfer resistance of the gas phase within the backing and the CL. The R_mt_ trend for the different MEA combinations is AQ(E79)/AQ20 < AQ(E79)/NF30 < NRE211/AQ20 < NRE211/NF30 under relative high humidity (70% RH) while AQ(E79)/AQ20 < NRE211/AQ20 < AQ(E79)/NF30 < NRE211/NF30 under dry condition (30% RH). It suggested that AQ(E79)/AQ20 has less mass transport problems, due to greater water management. The configuration of SSC membrane and SSC ionomer in CL has adequate water retention capability to prevent flooding issues under fully humidified conditions as well as effective water trapping in both membrane and CLs (excellent water uptake) to avoid MEA dehydration under dry conditions. As a result, either SSC membrane or SSC ionomer in CLs could reduce mass transport diffusion compared to its alternative LSC Nafion [34,35].

#### 3.6.4. Overall Resistance and Individual Contribution

The overall cell resistance is the sum of the membrane resistance (R_m_), the charge resistance (R_ct_), and the mass transfer (R_mt_). The R_m_ is mainly contributed to the cell ohmic resistance, the R_ct_ represents the charge transfer resistance (kinetic resistance) and the R_mt_ is more related to gas transport and water management. The individual resistance (ΔR_i_) as well as the overall resistances (ΣΔR_i_) of each MEA show a clear increase as RH decreased from 70% to 30%. The overall resistance ranking at 80 °C is AQ(E79)/AQ20 ≈ NRE211/AQ20 < AQ(E79)/NF30 ≈ NRE211/NF30 under wet condition (100%, 70%, and 50% RH, while AQ(E79)/AQ20 < NRE211/AQ20 < NRE211/NF30 ≈ AQ(E79)/NF30 under dry condition (30% RH), which could explain the performance difference of PLs in Figure 4.

**Figure 10:**
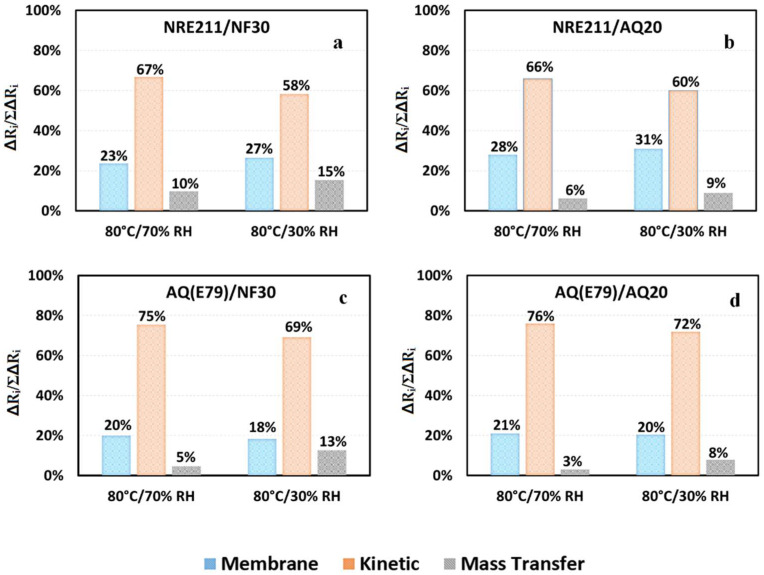
Relative contribution of each resistance to the overall resistance (ΔRi/ΣΔRi) for the selected MEAs (**a**) NRE-211/NF30, (**b**) NRE211/AQ20, (**c**) AQ(E79)/NF30, and (**d**) AQ(E79)/AQ20 at 80 °C under 70% and 30% of RH.

Studying the relative contribution of each resistance to the overall resistance increase could further elucidate the performance differences between these MEAs and provide some valuable insights for MEA design. Each component of this breakdown can be defined as ΔR_i_/ΣΔR_i_, in which R_i_ represents R_m_, R_ct_, or R_mt_. In Figure 10, the contribution of individual resistance is graphed under 70% and 30% RH, showing that R_ct_ comprises the majority of the total resistance and then followed by R_m_ and the least R_mt_. It suggests that the utilization of SSC ionomer as an alternative ionomer in CLs could bring more benefit than the substitution of LSC membrane with SSC membrane for the better cell performance. Moreover, when RH was reduced to 30%, the contribution of R_m_ for AQ (E79) based MEA went down, however for NRE211 based MEA, the portion of R_m_ in the total resistance increased by ~4%, indicating NRE211 experiences some dehydration under dry condition comparing to SSC membrane.

## 4. Conclusions

The fuel cell performance was evaluated and compared between the four MEAs with the combinations of SSC PFSA and LSC PFSA polymers as ionomers in CL and as membrane materials in MEAs, named as NRE211/NF30, NRE211/AQ20, AQ(E79)/NF30, and AQ(E79)/AQ20. According to the results of various electrochemical diagnoses such as ECSA, protonic resistance in CLs, H_2_ crossover, PLs, cell resistance through in-situ EIS measured at different RH% at 80 °C and 95 °C, it was found that SSC PFSA polymer used as membrane and ionomer in CL yields better fuel cell performance than LSC PFSA polymer, especially at high temperature and low RH conditions. Among the four investigated MEAs, AQ(E79)/AQ20 demonstrated the best cell performance at 80 °C and 95 °C especially under 30% of RH benefiting from the concurrence of both SSC membranes and SSC ionomer in CLs and the better compatibility effects using the same polymer material. The less mass transfer resistance of this MEA in EIS indicates a better water management with SSC polymer, which could avoid dehydration in either the membrane or catalyst layer under “dry” conditions (e.g., 30% RH) as well as mitigate catalyst layer flooding under “wet” conditions (e.g., 100% RH). Moreover, it suggests that SSC polymer as an ionomer in CLs could result in a more noticeable improvement in cell performance than SSC polymer as a membrane material in MEA, since the charge transfer resistance in the kinetic range contributed more to the overall cell resistance than the membrane resistance did.

## Figures and Tables

**Table 1:** List of the investigated MEAs.

Name of MEAs *	Membrane	Cathode CL	
		Ionomer	Content
NRE211/NF30	Nafion^®^ NR211	Nafion^®^ D-520	30%
NRE211/AQ20	Nafion^®^ NR211	Aquivion^®^ D83-15C	20%
AQ(E79)/NF30	Aquivion^®^ E79-03S	Nafion^®^ D-520	30%
AQ(E79)/AQ20	Aquivion^®^ E79-03S	Aquivion^®^ D83-15C	20%

* 30 wt% Nafion^®^ D-520 ionomer in all anode CLs.

**Table 2:** Electrochemical performance of the four investigated MEAs measured at 80 °C and 100% of RH.

MEAs	Electrochemical Surface Area ECSA (Desorption) (m^2^ g^−1^)	Double Layer Capacitances C_dl_ (mF cm^−2^)	H_2_ Crossover Current iH_X_ (mA cm^−2^)	Proton Resistance R_p_ (MEA) (mΩ cm^2^)	Tafel Slope (mV dec^−1^)
NRE211/NF30	38.0	20.3	1.60	99	67
NRE211/AQ20	37.5	19.6	1.58	163	65
AQ (E79)/NF30	39.6	19.0	1.24	109	70
AQ (E79)/AQ20	38.0	20.1	1.20	174	67

**Table 3:** Membrane resistances and relative conductivities calculated from both R_Membr_. and in-situ EIS at 1.0 A cm^−2^ at 80 °C under 70% RH vs. 30% RH.

MEAs	NRE-211/NF30	NRE-211/AQ20	AQ (E79)/NF30	AQ (E79)/AQ20
80 °C, 70% RH				
R_Membr_. (mΩ)	3.27 ± 0.03	3.15 ± 0.05	3.47 ± 0.02	2.94 ± 0.02
σ_R_ (mS cm^−1^)	34.25 ± 0.31	35.56 ± 0.56	38.04 ± 0.22	44.90 ± 0.31
R_m_ (mΩ)	2.42	2.48	2.11	1.95
σ_Rm_ (mS cm^−1^)	46.28	45.16	62.56	67.69
80 °C, 30% RH				
R_Membr_. (mΩ)	10.14 ± 0.07	8.62 ± 0.03	7.73 ± 0.02	5.90 ± 0.02
σ_R_ (mS cm^−1^)	11.05 ± 0.07	12.99 ± 0.05	17.08 ± 0.05	22.37 ± 0.07
R_m_ (mΩ)	5.86	5.76	4.00	3.68
σ_Rm_ (mS cm^−1^)	19.11	19.44	33.00	35.87

## Data Availability

Not applicable.

## Supplementary Materials

The following are available online at https://www.mdpi.com/article/10.3390/ma15010078/s1, Figure S1: Schematic diagram of membrane-electrode assembly (MEA). Figure S2: *iR*-compensated PLs for all the MEAs at 80 °C and various RH. Method: In-situ EIS.
