# Supplementary material for: Superior Proton Exchange Membrane Fuel Cell (PEMFC) Performance Using Short-Side-Chain Perfluorosulfonic Acid (PFSA) Membrane and Ionomer
Source: Materials (Basel). 2021 Dec 23;15(1):78. doi: 10.3390/ma15010078 (PMC8745893; doi:10.3390/ma15010078)
Supplement: Supplementary file 1 [file materials-15-00078-s001.zip › materials-1491792-supplementary.pdf]

materials

MDPI

Article

Superior Proton Exchange Membrane Fuel Cell (PEMFC) Performance Using Short-Side-Chain Perfluorosulfonic Acid (PFSA) Membrane and Ionomer

Nana Zhao, Zhiqing Shi *, Francois Girard

Energy, Mining & Environment Research Centre, National Research Council Canada, 4250 Wesbrook Mall, Vancouver, BC V6T 1W5, Canada; Nana.Zhao@nrc-cnrc.gc.ca (N.Z.); francois.girard@nrc-cnrc.gc.ca (F.G.)
* Correspondence: Zhiqing.Shi@nrc-cnrc.gc.ca

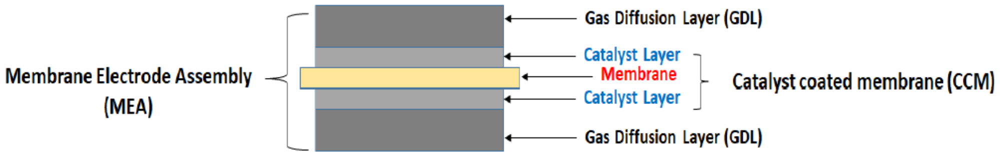

Figure S1. Schematic diagram of membrane-electrode assembly (MEA).

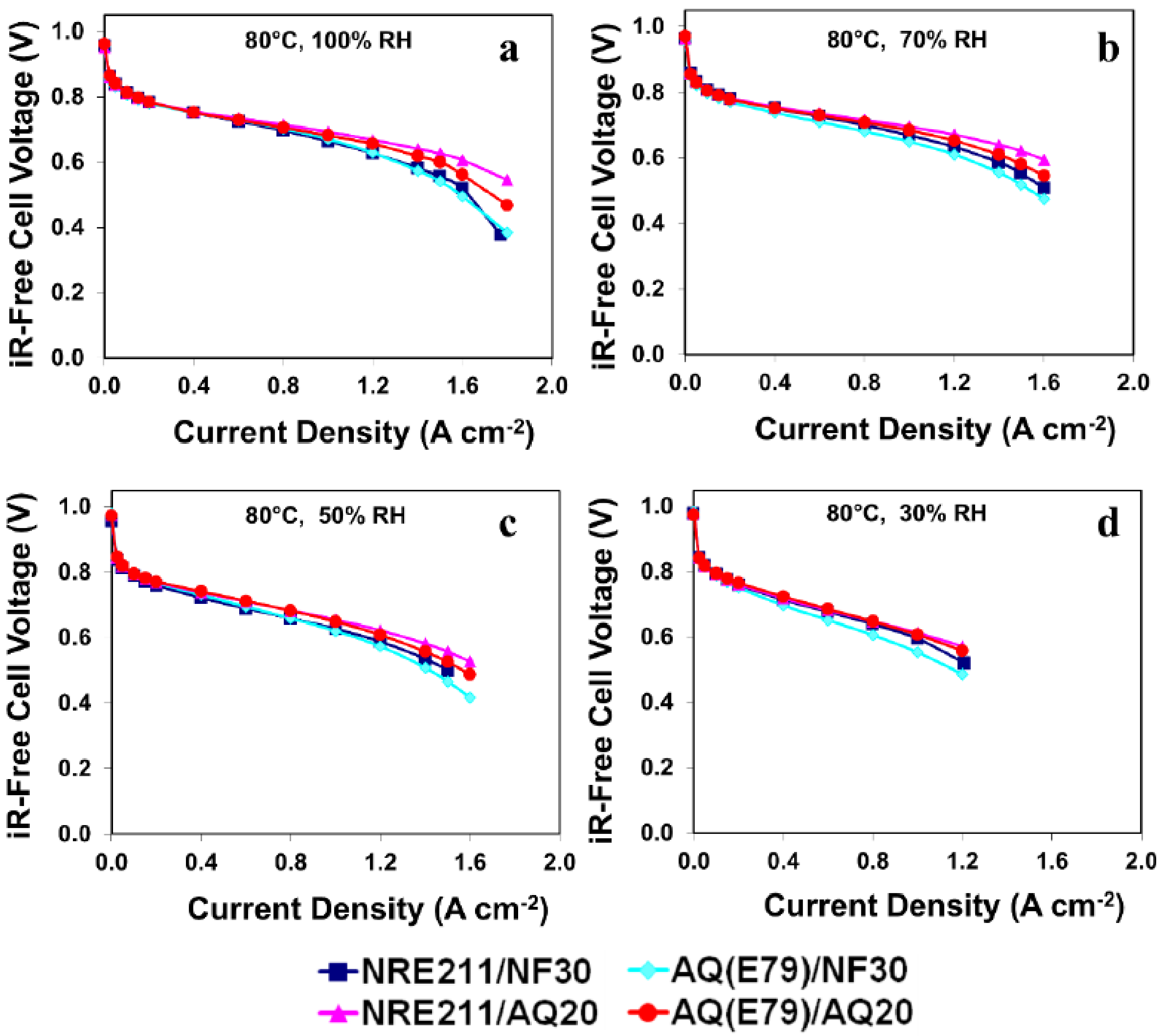

Figure S2. iR-compensated PLs for all the MEAs at 80°C and various RH.

Method: In-situ EIS

In-situ EIS was conducted during polarization curve collections (under constant direct current (DC)) by imposing an amplitude alternating current (AC) signal to the fuel cell via a load bank. The perturbation amplitude for the AC impedance was 5% of the direct current over a frequency range of 10 kHz to 0.1Hz. The voltage responses were recorded and decoupled by a built-in frequency response analyzer (FRA, Scribner 880).

Generally, when the cell is operated at high current densities or overpotential, the diffusion layer inside the MEA can cause the low-frequency end of the impedance to bend over the real axis, giving rise to a double semicircle. Figure 8 shows the spectra (Nyquist plots) of four MEAs, consisting of one high-frequency (HF) capacitive loop, one medium-frequency (MF) capacitive loop, and one low-frequency (LF) capacitive loop. The HF loop can be explained as either the electronic contact impedance between the electronic loop and the gas diffusion layer, or the ionic ohmic drop coupling with double-layer inside the active layer. This HF small loop can be fitted by a contact resistance R1 in parallel with a contact capacitance C1. The MF impedance arc is mainly attributed to the charge transfer resistance predominated by the effective charge-transfer resistance associated with the ORR, and the LF domain is mainly attributed to the mass transfer resistance of the gas phase within the backing and the catalyst layer. In accordance with the characteristics of fuel cell reaction and the observed electrochemical phenomena, the MF and LF loops can be fitted by a charge transfer resistance Rct in parallel with a constant phase element

(CPE1) and a mass transfer resistance Rmt in parallel with a constant phase element (CPE2), respectively. Rm represents the membrane resistance. The equivalent circuit for EIS fitting is shown in Figure 8 and the fitting of curves were carried out using the built-in impedance modeling software package, Z-view.
